# Parylene C as an Insulating Polymer for Implantable Neural Interfaces: Acute Electrochemical Impedance Behaviors in Saline and Pig Brain In Vitro

**DOI:** 10.3390/polym14153033

**Published:** 2022-07-27

**Authors:** Yuan Zhang, Jing Zhang, Song Le, Lan Niu, Jin Tao, Jingqiu Liang, Lihua Zhang, Xiaoyang Kang

**Affiliations:** 1 Laboratory for Neural Interface and Brain Computer Interface, Engineering Research Center of AI & Robotics, Ministry of Education, Shanghai Engineering Research Center of AI & Robotics, MOE Frontiers Center for Brain Science, State Key Laboratory of Medical Neurobiology, Institute of AI and Robotics, Academy for Engineering & Technology, Fudan University, Shanghai 200433, China; 19210860061@fudan.edu.cn (Y.Z.); 20210860066@fudan.edu.cn (J.Z.); 19210860075@fudan.edu.cn (S.L.); lihuazhang@fudan.edu.cn (L.Z.); 2 Ji Hua Laboratory, Foshan 528200, China; niulan@jihualab.com; 3 State Key Laboratory of Applied Optics, Changchun Institute of Optics, Fine Mechanics and Physics, Chinese Academy of Sciences, Changchun 130033, China; taojin@ciomp.ac.cn (J.T.); liangjq@ciomp.ac.cn (J.L.); 4 Yiwu Research Institute of Fudan University, Chengbei Road, Yiwu 322000, China; 5 Research Center for Intelligent Sensing, Zhejiang Lab, Hangzhou 311100, China

**Keywords:** Parylene, biocompatible polymer, electrochemical impedance, neural electrode, equivalent circuit model, saline, pig brain, in vitro

## Abstract

Parylene is used as encapsulating material for medical devices due to its excellent biocompatibility and insulativity. Its performance as the insulating polymer of implantable neural interfaces has been studied in electrolyte solutions and in vivo. Biological tissue in vitro, as a potential environment for characterization and application, is convenient to access in the fabrication lab of polymer and neural electrodes, but there has been little study investigating the behaviors of Parylene in the tissue in vitro. Here, we investigated the electrochemical impedance behaviors of Parylene C polymer coating both in normal saline and in a chilled pig brain in vitro by performing electrochemical impedance spectroscopy (EIS) measurements of platinum (Pt) wire neural electrodes. The electrochemical impedance at the representative frequencies is discussed, which helps to construct the equivalent circuit model. Statistical analysis of fitted parameters of the equivalent circuit model showed good reliability of Parylene C as an insulating polymer in both electrolyte models. The electrochemical impedance measured in pig brain in vitro shows marked differences from that of saline.

## 1. Introduction

Parylene, including a xylylene polymer family, is usually prepared by vacuum deposition polymerization. Parylene C (poly(2-chloro-*p*-xylylene)) was the first type to rate the USP class VI in the family, and it is impermeable to water and air, chemically inert, biocompatible, insulating and optical transparent [1]. Due to these excellent properties, it is widely applied to protect and insulate biomedical devices, such as neural interfaces [2], biomedical sensors [3,4] and implantable prostheses [5,6], as well as to modify culture substrates [7,8]. Implantable neural electrodes, the common tools for neural stimulation and neural recording, also require insulating materials, such as Parylene, as uniform coatings which provide insulation and protect against corrosion [9,10].

Characterization of neural electrodes can be conducted in various electrolyte models, such as normal saline [11,12,13,14], phosphate-buffered saline (PBS) [15,16,17] and interstitial fluid (ISF) [18,19], as well as neural tissue in vivo. Meanwhile, the performance of Parylene as an insulating polymer has been studied in these regular electrolyte solutions and in vivo [20,21]. Furthermore, electrochemical impedance spectroscopy (EIS) has been proved to be a feasible method to evaluate the properties of the polymer–metal system [20], which is also a key factor for the device microfabrication and electrochemical evaluation of neural electrodes.

In the context of electrochemical impedance for a neural electrode, what matters is the immediate liquid environment surrounding the electrode surface in vivo, which has many similarities with simple PBS [22]. The extracellular fluid (ECF) in the brain comprising Na+, Cl−, H+ and K+ species are comparable with that of PBS [23]. ISF further simulates the brain environment by containing a multitude of proteins. In this case, the immediate electrochemical performance, such as the electrochemical impedance which is related to the fundamental properties of the electrode, should only have minor differences between the salt electrolyte and pig brain in vivo. However, it is predictable that there is a huge gap between these salt electrolytes and neural tissue in vivo. The acute effect on the impedance of the neural electrode in the brain in vivo was more significant than that in the model protein solution, indicating that the protein absorption was not the only reason for the increased impedance [24]. Neural tissue contains not only biological fluids but also various types of neurons that have different chemical compositions and biological structures. These electrolytes, which can be seen as a kind of salt solution, are far from living tissues due to the anisotropy of tissues [25]. In terms of electrical properties, the resistance of neural tissue is 4–5 times that of cerebrospinal fluid [26]. A recent study investigated benchtop experiments in saline and in vivo in a single acute experiment in a pig brain and showed that the impedances of electrodes are consistently lower in saline as compared to that in brain tissue, particularly at the higher frequencies of interest (1 kHz–10 kHz) [27]. From this point of view, there is no big difference among these salt electrolytes for in vitro characterization, but the distinction between in vitro and in vivo characterization cannot be neglected.

Although ECF and salt electrolytes only have minor differences, the surrounding liquid environment of tissue in vivo is not as rich as that of salt electrolytes in vitro. Although about 80% of the brain is water, the water in the brain does not flow freely [28]. Less than 10% of the brain is ECF, and even less in gray matter [29]. Furthermore, when a neural electrode is immersed in salt electrolytes, the electrolyte fluid has full and homogeneous contact with the electrode together with the insulation layer. In contrast, while the neural electrode is implanted in the neural tissue, the ECF cannot perfectly contact the electrode because of the tissue cells, small vessels and the possible tiny space developed from the implantation. These differences may affect not only the working conditions of neural electrodes but also the insulation performance of coatings.

Furthermore, in vivo assessment involves animal preparation and experimental animal ethics, which is not convenient to access in the fabrication lab of neural electrodes. In comparison, animal brains in vitro are a kind of potential substitute, which are cheaply available from the slaughterhouse and easy to operate for implantation. Even if it is not as fresh as a brain that is just dissected, the chilled animal brain from supermarkets is an agricultural by-product that has the potential as a measurement environment. Here, we investigated the electrochemical impedance behaviors of Parylene C coating for a neural electrode both in normal saline and in a chilled pig brain in vitro. First, we prepared platinum (Pt) wire electrodes and performed EIS measurements both in the normal saline and pig brain in vitro. Then, the electrochemical impedance at the representative frequencies was statistically analyzed and discussed, which can help construct an equivalent circuit model. In particular, the equivalent circuit model was constructed and adjusted so that the source of electrochemical impedance was divided into three parts, namely the electrode–electrolyte interface, insulating layer and electrolyte. Additionally, we conducted a statistical analysis of the fitted parameters of the equivalent circuit model, which contributed to the comparability of the two electrolyte models and the characterization of Parylene C behaviors in the in vitro brain tissue.

## 2. Materials and Methods

### 2.1. Neural Electrode

**Figure 1:**
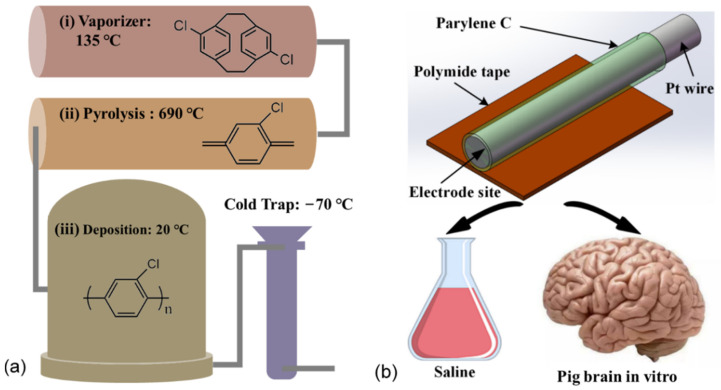
The schematic diagram of the experimental design. (**a**) The Parylene C coating was deposited by a chemical vapor deposition process. (**b**) The Pt wire electrodes were fabricated from a Pt wire with a diameter of 30 μm coated with 5 μm-thick Parylene C and then stuck to Polyimide tape for implantation. EIS measurements were performed for all the electrodes in both saline and pig brains in vitro.

Pt wire (30 μm in diameter) electrodes were used as the neural electrode. Firstly, the Pt wire was coated with 5 μm Parylene C by PDS 2010 (Public Laboratory for micro nanofabrication and devices, FUDAN University). Secondly, the Pt wire was stuck to a Polyimide tape with a thickness of 125 μm and a width of 4 mm. As a result, the Pt wire was reinforced and can be implanted in the pig brain by a clamping holder. Finally, the Pt wire was cut by surgical scissors to make an electrode site with a diameter of 30 μm. Additionally, the other side of the Pt wire was burned with a lighter to remove the coating of Parylene. The Pt wire was connected by a flat copper clip for the electrochemical measurements. Figure 1 is the schematic diagram of a Pt wire electrode.

### 2.2. Electrochemical Measurements

The electrochemical measurements were conducted in normal saline (0.9 % NaCl, pH 7.4) at room temperature with a standard three-electrode system, using an Ag/AgCl reference electrode and a titanium (Ti) wire (0.2 mm in diameter) counter electrode. On the other hand, the electrochemical measurements were also taken I n thepig brain with a standard three-electrode system, using Ti wires (0.2 mm in diameter) as the reference electrode and the counter electrode. The Ag/AgCl reference electrode used in saline is not suitable as a brain implant because it contains a salt electrolyte solution. On the other hand, the Ag/AgCl reference electrode used in saline is too big to implant inside the brain, as it can seriously damage the brain structure. The pig brain was ordered from the supermarket (Jinluo Shengxian, Shanghai, China), and is normally used as an ingredient for hotpot. The chilled pig brain was kept in a cold chain transport vehicle for two days. As a result, the as-received pig brain was fresh and soft. The pig brain was kept at room temperature for 1 h before being used for electrochemical measurements. Electrochemical impedance spectroscopy (EIS) measurements were performed on a PGSTAT302N electrochemical workstation (AUTOLAB) by sending a voltage sinewave of 100 mV amplitude within the frequency range from 1 Hz to 100 kHz. The EIS was first taken in normal saline, and then the Pt wire electrode was inserted into the pig brain for the next EIS measurement. The placements of Pt wire electrodes were not unified for all measurements. We did not repeat the implantation in the same location, as this may result in tissue damage at the implanted site. In this experiment, 42 electrodes took the EIS measurement. It took about 2.5 h for the measurement in the pig brain model.

### 2.3. EIS Analysis

The EIS data were analyzed by RelaxIS (version 3.0.14, rhd instruments GmbH & Co. KG, Darmstadt, Germany). RelaxIS is a rich software suite that combines many tools used for the evaluation of spectra measured in impedance spectroscopy experiments. The equivalent circuit model was also built and fitted in RelaxIS. The data at the frequency of power-line interference and bad data points were removed before fitting. The CDF (cumulative distribution function) plots were used to check the validity of a fit. The CDF defines the probability that, according to the given distribution, a randomly chosen value X is less than or equal to the function value x. If a model correctly describes measured data, the deviations between model and fit should be due to normally distributed noise. Three of the forty-two measured data were removed from the further analysis. The three measured data are greatly different from other data in the Bode diagrams and thus have poor fitting performance, so the mismatch may be caused by the electrode itself rather than the fitting model.

The electrochemical impedance at the representative frequencies was analyzed to help construct an equivalent circuit model. The impedance at 1 kHz is widely referred to to evaluate neural electrodes. Furthermore, the impedance of electrodes at high frequency (100 kHz) describes the characteristics of capacitance components, while the impedance at low frequency (1 Hz) describes the characteristics of other components, such as resistance. In addition, the impedance at 12 Hz characterizes the electroactive area of electrodes [30,31,32].

Impedance angle refers to the phase difference between the applied voltage and current in an AC circuit. It is also very useful for building the equivalent circuit model. Pure capacitance shows an impedance angle of −90°, while pure resistance shows an impedance angle of 0°. If the impedance angle is between −90° and 0°, the circuit shows the properties of both the capacitance and resistance. When the impedance angle is close to −90°, it shows more overall capacitance property. If the impedance angle is close to 0°, it shows more overall resistance property.

To better understand the contribution of each part in the electrode–electrolyte system in the EIS, the equivalent circuit model was constructed. Based on the methods reported earlier [33,34,35] and the EIS at different frequencies, a constant phase element (CPE) was used in the EIS analysis of Pt wire neural electrodes. The CPE describes a non-ideal capacitance, expressed by the equation:

(1) $$\hat{Z}=\frac{1}{Q\cdot {\left(i\omega \right)}^{\alpha }}$$

where Q is the CPE’s admittance value, alpha is the CPE’s exponential factor, ω is the angular frequency (rad s^−1^) = 2 πf and f is the frequency in Hz. If alpha is 1, the CPE is an ideal capacitor with a phase angle of −90°, while for lower values of alpha, the phase angle is lowered to −(90° * alpha). The parameter changed from 0 to 1, corresponding to the CPE, from a pure resistance to a pure capacitance.

To evaluate the difference between the spectra in saline and brain in vitro, both electrochemical impedance at the representative frequencies and the fitted parameters of the equivalent circuit model were statistically analyzed. For all the comparisons between the saline and the brain group, normality tests of the data collected from different electrodes were first manipulated in each group using Shapiro–Wilk tests. The data from both groups that were likely to be normally distributed were subjected to one-way analysis of variance (ANOVA), while others were subjected to Kruskal–Wallis analysis of variance. The significance level was set at * *p* < 0.05; ** *p* < 0.01. To demonstrate the comparison, the impedance value measured and the fitted parameter of the equivalent circuit model were presented by box-whisker plots, where the boxes indicate 25% to 75% of each group, the whiskers are the upper and lower boundaries which are in the ±1.5× interquartile range, the blue dots represent the average and the red and green diamonds represent the raw data of the saline and pig brain groups, respectively.

## 3. Results

### 3.1. Electrochemical Impedance Spectroscopy (EIS)

**Figure 2:**
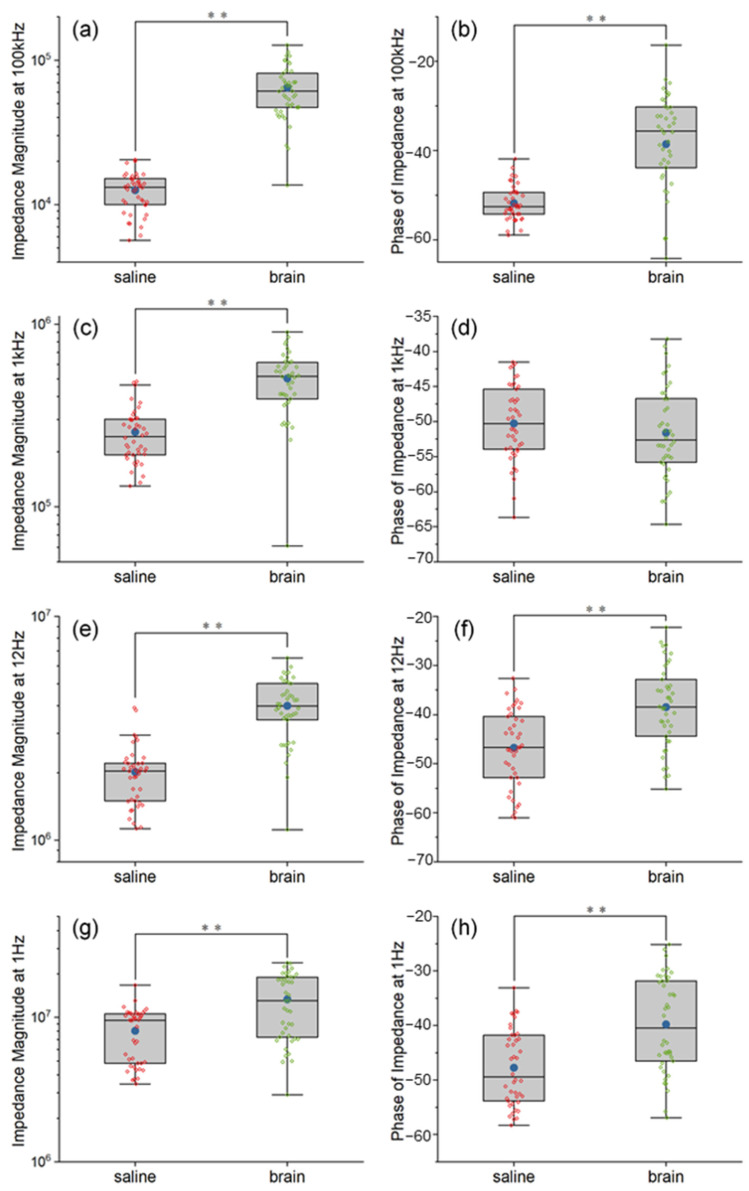
The comparison between the impedance of Pt wire neural electrodes in saline and pig brain in vitro at 100 kHz (**a**,**b**), 1 kHz (**c**,**d**), 12 Hz (**e**,**f**) and 1 Hz (**g**,**h**). Significance level was set at ** *p* < 0.01.

Figure 2 show the comparison between the impedance of Pt wire neural electrodes in saline and pig brain in vitro. The exact impedance values in both saline and pig brain models at the representative frequencies and the statistical report of the comparison between the two models are provided in Tables S1 and S2, respectively. Overall, the impedance magnitudes from the EIS measurements in the pig brain model were larger compared with that of saline, and the impedance phases in the pig brain were also larger except at 1 kHz. The detailed results were described as follows.

The impedance magnitudes at 100 kHz increased significantly after being implanted inside the pig brain, shown in Figure 2a (F = 167.33, *p* < 0.01). The mean and the median of impedance magnitudes inside the pig brain were 5.14 and 4.64 times larger than that of saline, respectively. The explanation is that the pig brain environment was less conductive than the saline environment, which matched the results measured in vivo [26,36]. Figure 2b show the impedance phases at 100 kHz, which were between −90° and 0° as a behavior of capacitance. These features indicated that the non-ideal capacitance was added at an extremely high frequency for the electrode both in the saline and pig brain. Compared with the pig brain environment, the saline environment showed more capacitance properties for the Pt wire neural electrode.

The impedance at 1 kHz is commonly compared as a mark of the neural electrodes. As we can see, the mean and the median of impedance magnitudes were increased by 1.96 and 2.13 orders after the electrodes were implanted in the pig brain, which is shown in Figure 2c. This can be explained by the high diffusion resistance in pig brain tissue, which was due to the restriction of ion movement by neurons around the electrode site. Based on the hydrogen atom deposition and desorption reaction between the water and Pt surface as shown in Equation 1, the interface resembled a capacitive reaction as the so-called “pseudocapacitive charge injection” mechanism [18,37].

(2) $$\mathrm{Pt}+H_{2}O+e^{-}↔\mathrm{Pt}-H+{\mathrm{OH}}^{-}.$$

Thus, the impedance phases of the Pt wire neural electrode inside the saline and pig brain showed comparable capacitance properties because both of them are neutral environments, as shown in Figure 2d. It can be explained as follows: In this experiment, the pig brain was fresh and thus had a similar quality to the neural tissues in vivo. Additionally, both the saline and the pig brain can be seen as neutral electrolyte environments. The pseudo-capacitance of the Pt electrode is mainly related to the hydrogen atom and OH- in the electrolyte. There is no difference between the water in the pig brain and the salt solution, and both electrolytes are neutral. Therefore, the pseudo-capacitance in the two electrolytes is close to each other. In terms of the equivalent circuit model, 1 kHz is a frequency that allows current to pass through all the elements in the circuit (including the constant phase element that refers to pseudo-capacitance), so the phase at 1 kHz is dominated by the phase of the pseudo-capacitance. Based on the inference of the comparable pseudo-capacitance, it can be understood that the phase at 1 kHz is also comparable.

The impedance at 12 Hz is also used to assess the electroactive area of the electrodes [30,31,32]. The impedance magnitudes of the pig brain environment at 12 Hz were larger than that of the saline environment, as shown in Figure 2e. The mean and the median of impedance magnitudes inside the pig brain were 1.98 and 1.95 times larger than that of saline, respectively. That means the electroactive areas of the Pt wire neural electrode inside the pig brain environment are smaller than that of the saline environment (χ^2^ = 44.30, *p* < 0.01). However, the impedance phases of the saline environment still showed more capacitance properties than that of the pig brain environment at 12 Hz, as shown in Figure 2f. The reason is that the pseudocapacitive charge injection mechanism of the Pt wire neural electrode is faster in the saline environment.

The impedance at the extremely low frequency (1 Hz) is considered to show the dominating resistance properties of the electrodes. The impedance magnitudes of the pig brain environment at 1 Hz were larger than that of the saline environment (χ^2^ = 15.07, *p* < 0.01), as shown in Figure 2g, which indicated that the dominating resistance parts in the pig brain environment were larger than those in a saline environment. The mean and the median of impedance magnitudes inside the pig brain were 1.65 and 1.36 times larger than that of saline, respectively. However, as shown in Figure 2h, the saline environment showed more capacitance properties at 1 Hz, which also indicated the double layer capacitance of the Pt electrode–electrolyte interface in a pig brain environment was smaller than that in a saline environment.

### 3.2. Equivalent Circuit Model Based on EIS

**Figure 3:**
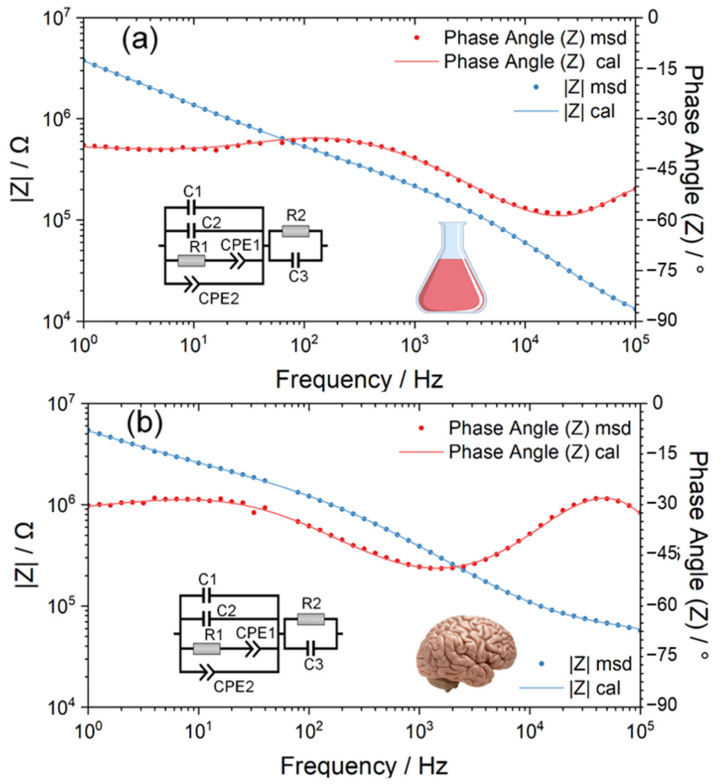
The electrochemical impedance spectroscopy (EIS) of the Pt wire neural electrodes was measured in normal saline (**a**) and pig brain in vitro (**b**), and the inserted diagrams are their corresponding equivalent circuit models.

Based on the EIS discussion at different frequencies, an equivalent circuit model was constructed, as shown in Figure 3. The circuit model of the Pt electrode–electrolyte interface consists of a double layer capacitance C1 in parallel with its diffusion resistance R1 and a constant phase element CPE1 representing the pseudo-capacitance of Pt electrodes. The Parylene C layer is represented by an inherent capacitance of Parylene C2 in parallel with its constant phase element CPE2 characterizing the leakage properties, which is also in parallel with that of the neural electrode. Here, the two plates of the inherent capacitance of Parylene are the Pt wire and saline/pig brain electrolyte. Additionally, the Parylene layer is the dielectric layer. Thus, here, the inherent capacitance of Parylene means the capacitance across the Pt wire, Parylene layer and the saline/pig brain electrolyte. The R2 and C3 are the resistance and capacitance of the saline and the pig brain, respectively.

Based on the measured EIS data, the representative numerical fitting results of the equivalent circuit of Pt wire neural electrodes both inside the saline and pig brain are shown in Figure 3. As displayed in Figure 3, the equivalent circuit model provides a fairly good fitting to measured EIS data as the χ^2^ is 4.5942 × 10^−5^ and 6.6699 × 10^−5^ for the measurement in saline and pig brains, respectively. Table S3 show the statistical fitted parameters of the equivalent circuit model in the saline and the pig brain model, and Table S4 provide the statistical report of the comparison between the two models.

### 3.3. Statistical Analysis of Fitted Parameters of Equivalent Circuit Model

#### 3.3.1. Pt Electrode-Electrolyte Interface

**Figure 4:**
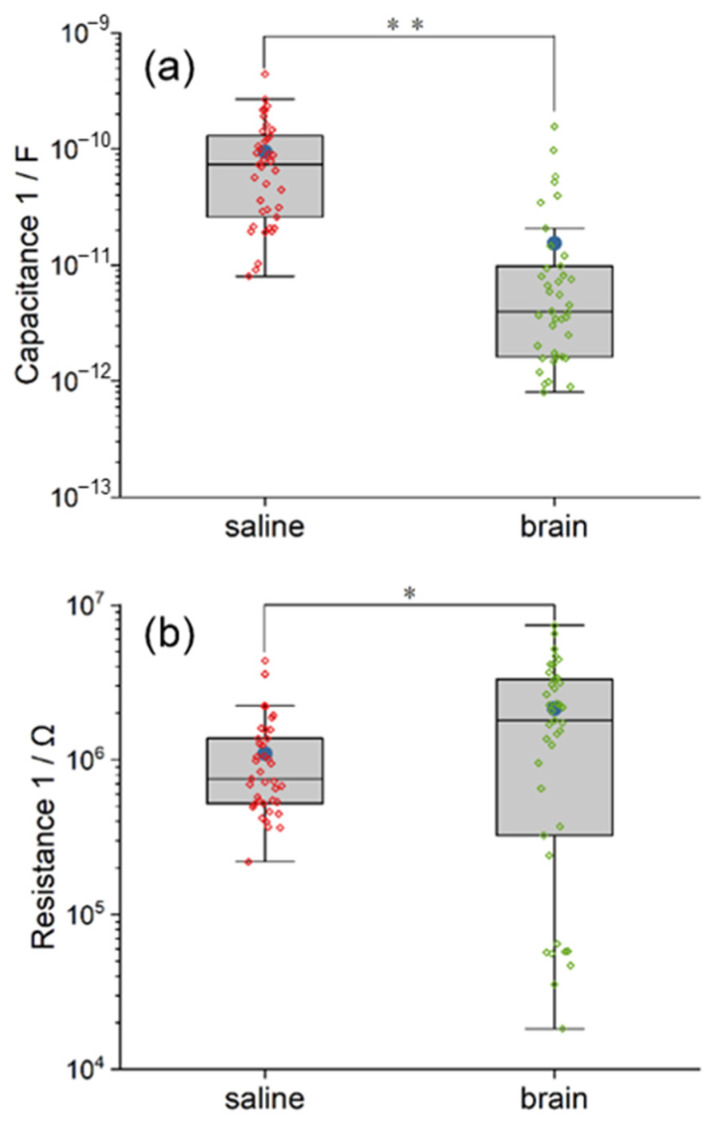
The comparison between the equivalent circuit model of saline and pig brain in the fitted value of C1 (**a**), R1 (**b**). The differences in C1 and R1 between the two models indicate the impact of electrolytes on the double layer capacitance and the diffusion resistance. Significance level was set at * *p* < 0.05; ** *p* < 0.01.

**Figure 5:**
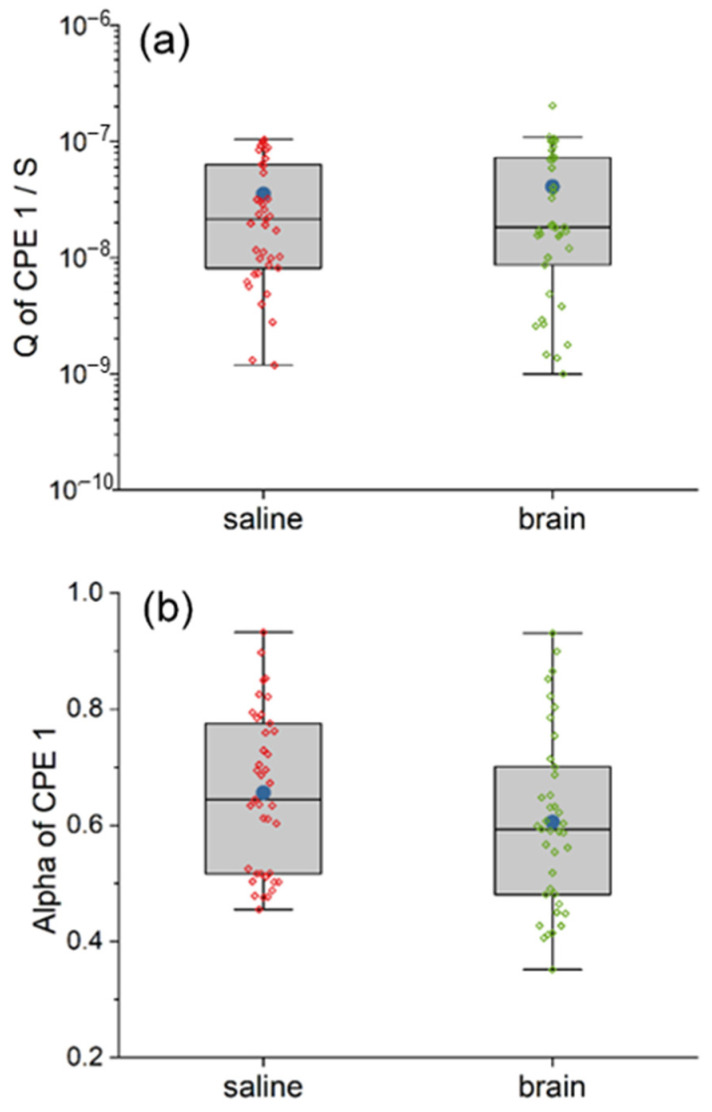
The comparison between the equivalent circuit model of saline and pig brain in the fitted value of Q (**a**) and alpha (**b**) of CPE1. The CPE1 in the two models are similar, indicating similar pseudo-capacitance.

The capacitance C1 (double layer capacitance), the resistance R1 (diffusion resistance) and the constant phase element CPE1 were assembled to make up the circuit model of the Pt electrode–electrolyte interface. Here, we used the CPE to characterize the pseudocapacitive charge injection mechanism of the Pt electrode–electrolyte interface. Figure 4 and Figure 5 are the box plots of the fitted value of C1, R1, Q and alpha of CPE1, respectively, together with the mean (plotted in blue dot) and all the raw data (plotted in red or green diamond).

As demonstrated in Figure 4a, more values of fitted C1 in the circuit model of pig brain are outliers. Both the mean and the median of double layer capacitance C1 in the saline model are larger than those in the pig brain model, and the statistical difference between the two groups is very significant (χ^2^ = 39.07, *p* < 0.01). The mean and the median capacitance C1 inside the saline are 6.51 and 7.20 times larger than that of the pig brain, respectively. The reason is that the saline environment is more liquid and fluid than the pig brain environment.

According to Figure 4b, the fitted values of diffusion resistance R1 in the model of pig brain are more scattered, of which the difference between the maximum and the minimum is approximately 2.6 orders of magnitude. However, it can still be determined that the mean and the median of R1 in the pig brain model are larger in comparison with those in the saline model, and the statistical difference between the two groups is significant (χ^2^ = 4.26, *p* < 0.05). The mean and the median resistance R1 inside the pig brain are 1.95 and 2.38 times larger than that of saline, respectively. This result indicates that the charges around the Pt wire neural electrode were more difficult to diffuse in the pig brain environment.

As for pseudo-capacitance CPE1, the two models are not statistically different in both the Q and the alpha, which are shown in Figure 5. The mean and the median Q of CPE1 inside the pig brain are 1.16 and 0.86 times compared with that of saline, respectively, while both the mean and the median alpha of CPE1 inside the pig brain are 0.92 times compared with that of saline, respectively.

#### 3.3.2. Parylene C Layer

**Figure 6:**
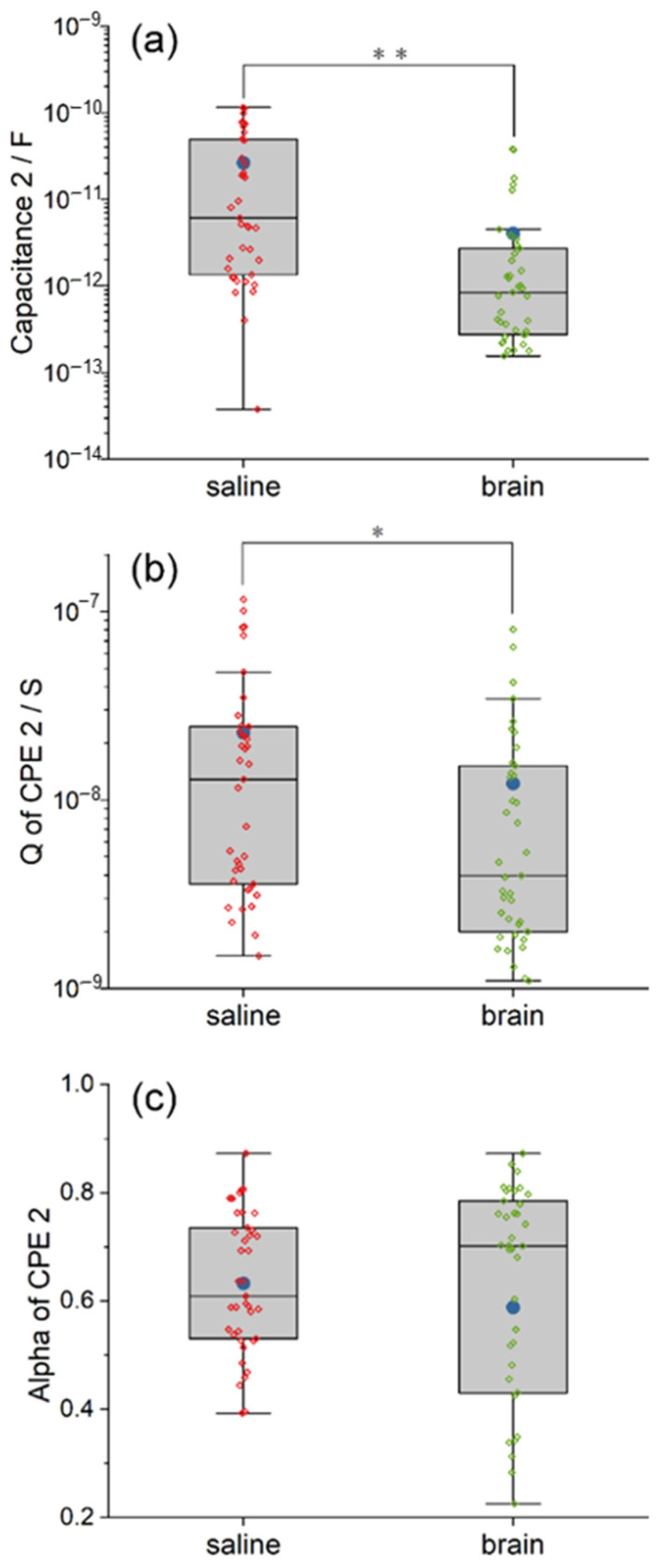
The comparison between the equivalent circuit model of saline and pig brain in the fitted value of C2 (**a**), Q (**b**) and alpha (**c**) of CPE2. The difference in C2 and CPE2 indicates that the Parylene C was in different states. Significance level was set at * *p* < 0.05; ** *p* < 0.01.

The model of the Parylene C layer includes the capacitance C2 (the inherent capacitance of Parylene) and its constant phase element CPE2. Here, we use the CPE2 to characterize the leakage properties of the Parylene C layer. A comparison between the equivalent circuit model of saline and pig brain in the fitted value of C2 and Q and alpha of the CPE2 is also illustrated in the box plots, as shown in Figure 6.

The fitted C2 in the circuit model of the pig brain is more concentrated but with some outliers (Figure 6a). The mean of C2 in the pig brain model is much higher than the median due to the outliers. According to the statistical analysis, the inherent capacitance of Parylene C2 in the saline model is significantly larger than that in the pig brain model (χ^2^ = 21.83, *p* < 0.01). The mean and the median capacitance C2 inside the saline are 1.95 and 18.4 times larger than that of the pig brain, respectively. We suggest the reduced overlap area between the pig brain and the Pt wire plays a key role in this result.

The behavior of CPE’s admittance Q is illustrated in Figure 6b. As we can see, the degree of dispersion of the Q of CPE2 is comparable in the two models, while the mean and the median in the saline model are relatively larger. Additionally, the difference is statistically significant (χ^2^ = 5.97, *p* < 0.05). The mean and the median Q of CPE2 inside the saline are 1.86 and 3.25 times larger than that of the pig brain, respectively. The smaller admittance in the pig brain indicated the smaller leakage properties of the Parylene C layer compared with that of saline. Although the alpha of CPE2 in the pig brain model is quite dispersed from 0.2253 to 0.8726, as shown in Figure 6c, there is no significant difference between the two models in the alpha of CPE2 (χ^2^ = 0.88, *p* > 0.05). The mean and the median alpha of CPE2 in the saline are 1.07 and 0.87 times larger than that in the pig brain, respectively. The results indicated that the tendency of leakage properties of the Parylene C layer from resistance to capacitance was not altered.

#### 3.3.3. Saline and Pig Brain as an Electrolyte

**Figure 7:**
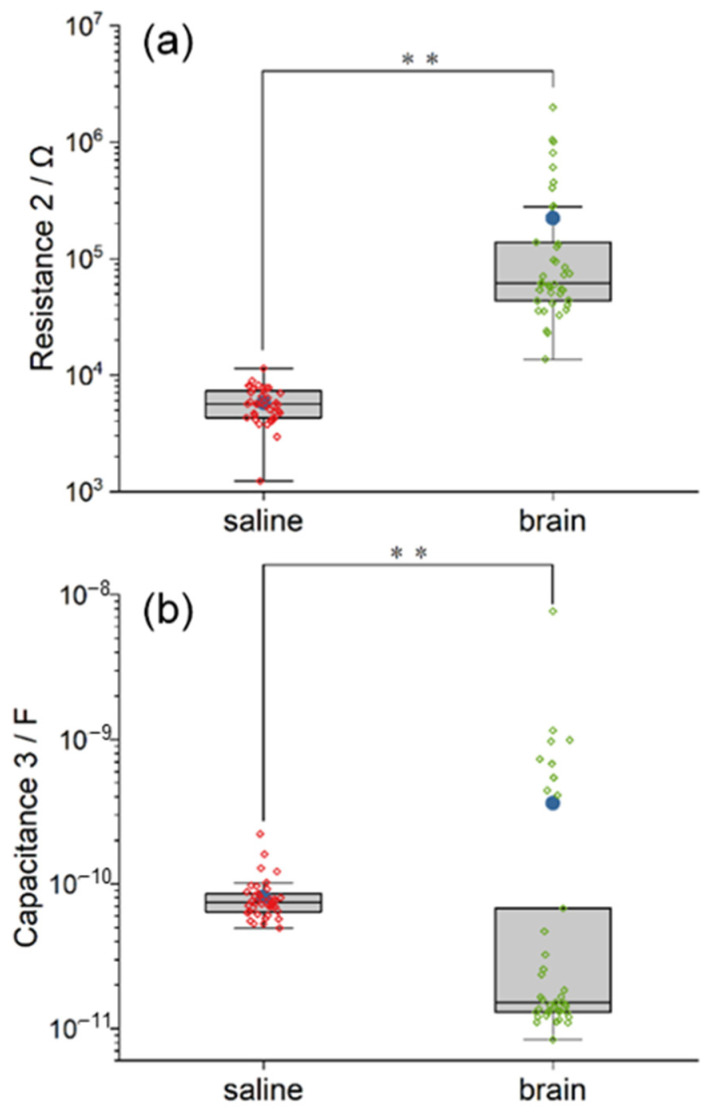
The comparison between the equivalent circuit model of saline and pig brain in the fitted value of R2 (**a**) and C3 (**b**). The saline model and the pig brain model differ in both capacitance and resistance. Significance level was set at ** *p* < 0.01.

The model of the electrolyte includes the resistance R2 and its capacitance C3. The illustrations of R2 and C3 are similar to the other parameter mentioned above. As demonstrated in Figure 7, the R2 and C3 in the saline model are highly clustered, while those in the pig brain model are dispersed with quite a lot of outliers. The mean and the median resistance R2 inside the pig brain model are 38.51 and 10.85 times larger than that in saline, respectively. The value of C3 in the pig brain model even develops into two clusters. The mean and the median capacitance C3 inside the pig brain are 4.45 and 0.2 times compared with saline, respectively. Furthermore, the statistical difference between the two models in both R2 and C3 is significant when *p* < 0.01 (χ^2^ _R2_ = 57.76, χ^2^ _C3_ = 15.86). Based on the equivalent circuit model, the results in R2 and C3 indicated the huge difference between saline and pig brains in electrical properties.

## 4. Discussion

In this work, the electrochemical impedance behaviors of Parylene C coating for a neural electrode were investigated both in normal saline and in a chilled pig brain in vitro. Saline is a simple in vitro electrochemical testing environment for neural electrodes. In comparison to an experimental animal, a chilled animal brain is convenient to access in the fabrication lab of neural electrodes in that it is cheaply available from the slaughterhouse and easy to operate for implantation. Although an in vitro test is not a substitute for an in vivo one, a chilled brain in vitro could facilitate the characterization of electrodes that are still in the preliminary stages of design. Additionally, we performed the EIS measurements in these two experimental environments.

Based on the EIS measurement, the impedance in the pig brain in vitro was markedly larger than that in saline. The difference in impedance may result from the huge gap in composition and structure between saline and tissues. The rich surrounding liquid environment in the salt electrolyte can lead to full water contact with the polymer coatings [20]. In contrast, while the neural electrode is implanted in the neural tissue, the ECF cannot perfectly contact the polymer coatings because of the tissue cells. It was reported that the impedance amplitude of a 50-μm-diameter Pt electrode measured in vivo was greater than that in PBS for the frequency from 1 Hz to 100 kHz [38]. Similar results of neural electrodes made of different materials have also been reported [27,39,40,41,42]. The phase of impedance in the pig brain was comparable to that in saline at 1 kHz and less negative than that in saline at frequencies lower than 1 kHz, which was also similar to the measurement in vivo from the literature [27,40]. Based on the equivalent circuit model constructed, the contribution of the electrode–electrolyte interface, the insulating layer and the electrolyte to EIS can be discussed.

The results of statistical analysis on C1 of the equivalent circuit model indicated that the double layer capacitance at the Pt electrode–electrolyte interface was quite distinct due to the type of electrolyte (saline and pig brain). The diffusion resistance at the interface, represented by R1, was also influenced by different electrolytes. The result of CPE1 can be explained as the pseudo-capacitance that originated from the hydrogen atom deposition and desorption reaction and is similar both in the saline and pig brain environments. The amount of hydrogen atoms involved in the reaction is extremely small; therefore, the saline and pig brain environments make no difference.

As for the contribution of the Parylene layer, the capacitance and CPE’s admittance in the pig brain model was significantly reduced compared to that in the saline model, which is probably due to the reduced overlap area between the electrolyte and the Parylene layer. The capacitance is proportional to the area of overlap of the two plates. Here, the two plates of the inherent capacitance of Parylene are the Pt wire and saline/pig brain electrolyte. Since the Pt wire is the same in both of the electrolytes, the drop of C2 in the pig brain means the overlap area between the pig brain and Pt wire is decreased. When the Pt wire neural electrode is immersed in the saline electrolyte, the Parylene layer is well surrounded by the saline. The overlap area between the saline and Pt wire reaches its maximum. As for the pig brain electrolyte, the Pt wire neural electrode must push away the brain tissue to implant itself inside. In this case, there is always some tiny space between the brain tissue and the Parylene layer. Therefore, the overlap area between the pig brain and Pt wire is reduced compared with that of saline. Furthermore, the reduced overlap area in the pig brain model did not alter the overall tendency of leakage properties of the Parylene C layer from resistance to capacitance compared with that of saline, which resulted in the similarity of CPE2′s alpha in the two models.

According to the fitting results shown in Table S3, the value of C2 was in a similar order of magnitude to that of C1. Considering the physical meaning of the equivalent circuit model, the inherent capacitance of Parylene had a considerable impact on the double layer capacitance of the Pt electrode–electrolyte interface. The reason is that the diameter of the Pt wire neural electrodes in this experiment was only 30 μm in diameter so the double layer capacitance of the interface was reduced to a value similar to that of Parylene C. In addition, the capacitance and leakage of the Parylene C layer were also influenced by the type of electrolyte in which the pig brain cannot have thorough contact with the Pt wire neural electrode.

The resistance and capacitance of the two electrolytes can be expressed by R2 and C3, respectively. The resistance in saline was smaller than that in the pig brain, which matched the results in previous studies [36]. The results also explained the consistency of the value in the saline model. To be specific, saline provided a stable and homogeneous electrochemical environment for EIS tests; in addition, R2 and C3 had less relationship with the properties of the electrodes, unlike the other fitted parameters in the circuit model. On the other hand, the reason for the dispersity of the value in the pig brain model can be that the pig brain was a complex system with a specific structure. In particular, the pig brain has the anisotropic properties of resistance and capacitance. It has been reviewed that the resistivity of gray matter is smaller than that of white matter in both living tissues and tissue samples [36], and the anisotropy of neural tissues varies with the difference in physiological structures [43]. Hence, the scattered values in the pig brain model may be referred to as the differences in the composition and structure of the brain tissue between the electrodes which were caused by the inconsistent location of the implantation.

One obstacle faced by measurements in biological tissues is instability. Tissue conductivity can be influenced by several factors, including temperature as well as the time after death [25,36]. Additionally, different species vary in brain conductivity [44]. Hence, further studies are required to verify the reliability of the in vitro model. Furthermore, the value of C3 split into two clusters where the difference was approximately two orders of magnitude. This was possible because the placement of the working electrodes was not fixed relative to the counter electrode. The capacitance of the pig brain should be quite different when the working electrodes are implanted on the same or the other hemisphere due to the distance and electrochemical environment.

We speculated that the in vitro brain model could narrow the gap of electrochemical impedance between the in vitro electrolyte solution and neural tissue in vivo, which would be helpful in device fabrication and evaluation. However, there are limitations to the current study. Firstly, there was little foreign body reaction in the chilled pig brain model after implantation. Foreign body reaction has an impact on the condition of chronically implanted neural electrodes, even leading to failure of the implant [45]. Based on in vivo two-photon microscopy, microglia cell body movement was neglectable in the first 6 h after insertion [46], and oligodendrocyte precursor cells did not respond to injury within the first 12 h [47]. It was also reported that the extension of microglia did not reach the implant by the first-hour post-insertion [48,49]. According to the neural calcium imaging, the activity of neurons was kept to the baseline in 6–25 min post-implant, and the neurites showed axonal injury at 1–3 h [50]. Reactive astrocytes were detected as small clusters around the implant 1 day post-insertion [51]. However, the impedance spectrum in vivo varied in days after implantation [49,52]. The local neurodegenerative state was progressive for several weeks [53,54,55], and an astrocyte sheath was observed at 4 weeks post-insertion [51]. Therefore, although the measurement in the brain in vitro can reflect electrode performance in tissues in acute tests, in vivo characterization is still necessary. Secondly, the comparison between the EIS measurements in brain tissues in vitro and that in vivo was not performed in this work. It requires further studies of how different the brain would be in vitro and in vivo.

## 5. Conclusions

A chilled pig brain model was proposed as an in vitro electrolyte for the characterization of neural electrodes. We investigated the insulation behaviors of Parylene C coating in a chilled pig brain in vitro by measuring EIS together with the equivalent circuit model, which has not been reported previously. According to the measurements, the electrochemical impedance measured in the pig brain in vitro showed a marked difference from that in saline, and similar results measured in vivo were reported [38]. Analysis of fitted parameters of equivalent circuit model based on EIS provided a quantitative assessment of neural electrodes along with the electrode–electrolyte interface, the insulating layer and the electrolyte, which generated a better understanding of the behavior of Parylene. Although the pig brain model cannot replace the measurement in the saline model for neural electrodes, this brain model in the characterization process provides a complementary route for the electrochemical impedance assessment of neural implants in the lab. It can also contribute to the neural electrodes on both the microfabrication and electrochemical impedance evaluation, which is helpful in the translational research of neural electrodes. It is still too early to say that a pig brain in vitro can mimic the pig brain in vivo as an electrolyte since various factors impact the electrical properties of brain tissue, including species, temperature and time after death [25,36,44]. The comparison between the EIS measurements in vitro and in vivo requires further studies.

## Data Availability

The data presented in this study are available on request from the corresponding author.

## Supplementary Materials

The following supporting information can be downloaded at: https://www.mdpi.com/article/10.3390/polym14153033/s1; Table S1: The statistical values of electrochemical impedance in both saline and pig brain models at the representative frequencies; Table S2: The statistical report of the comparison between electrochemical impedance (magnitude and phase) in the saline and the pig brain model at the representative frequencies; Table S3: The statistical fitted parameters of equivalent circuit model in both saline and pig brain model; Table S4: Statistical report of the comparison between electrochemical impedance (magnitude and phase) in the saline and the pig brain model at the representative frequencies.
