# Supplementary material for: Parylene C as an Insulating Polymer for Implantable Neural Interfaces: Acute Electrochemical Impedance Behaviors in Saline and Pig Brain In Vitro
Source: Polymers (Basel). 2022 Jul 27;14(15):3033. doi: 10.3390/polym14153033 (PMC9332801; doi:10.3390/polym14153033)
Supplement: Supplementary file 1 [file polymers-14-03033-s001.zip › polymers-1788896-supplementary.pdf]

Table S1. Statistical values of electrochemical impedance in both saline and pig brain model at the representative frequencies.

	Electrolyte	Mean	Standard deviation (S.D.)	Min.	First quartile (Q1)	Median	Third quartile (Q3)	Max.
Magnitude at 100 kHz (kΩ)	saline	12.58	3.68	5.64	10.02	13.16	15.13	20.47
	brain	64.69	25.84	13.64	47.13	61.09	81.13	127.51
Phase at 100 kHz (°)	saline	-51.81	3.94	-58.96	-54.28	-52.63	-49.37	-41.86
	brain	-38.72	12.68	-84.75	-43.82	-35.86	-30.30	-16.34
Magnitude at 1 kHz (kΩ)	saline	256.32	88.73	129.81	192.35	242.27	301.13	484.13
	brain	503.74	177.92	61.09	388.72	516.01	616.02	903.56
Phase at 1 kHz (°)	saline	-50.22	5.50	-63.67	-53.96	-49.60	-45.39	-41.52
	brain	-51.52	6.51	-64.67	-55.81	-52.34	-46.75	-38.21
Magnitude at 12 Hz (kΩ)	saline	2016.67	2016.67	2016.67	2016.67	2016.67	2016.67	2016.67
	brain	2016.67	2016.67	2016.67	2016.67	2016.67	2016.67	2016.67
Phase at 12 Hz (°)	saline	-46.86	7.85	-61.04	-52.83	-46.69	-40.36	-32.62
	brain	-38.43	8.48	-55.14	-44.42	-38.32	-32.85	-22.20
Magnitude at 1 Hz (kΩ)	saline	8056.29	3263.49	3462.48	4806.73	9535.34	10547.00	16732.50
	brain	13322.30	6237.07	2911.10	7297.37	13023.00	18981.90	23885.30
Phase at 1 Hz (°)	saline	-47.70	7.02	-58.33	-53.86	-48.94	-41.79	-33.10
	brain	-39.73	8.82	-56.93	-46.54	-38.21	-31.82	-25.13

Table S2. Statistical report of the comparison between electrochemical impedance (magnitude and phase) in the saline and the pig brain model at the representative frequencies. Degree of Freedom = 1.

	F-value	χ^2-value	p-value
Magnitude at 100 kHz	167.33427	-	0
Phase at 100 kHz	-	33.43554	7.36654E-9
Magnitude at 1 kHz	-	40.00392	2.53454E-10
Phase at 1 kHz	0.95183	-	0.3322
Magnitude at 12 Hz	-	44.3006	2.8163E-11
Phase at 12 Hz	21.83147	-	1.18607E-5
Magnitude at 1 Hz	-	15.07451	1.03349E-4
Phase at 1 Hz	-	14.77694	1.21006E-4

Table S3. The statistical fitted parameters of equivalent circuit model in both saline and pig brain model

	Electrolyte	Mean	Standard deviation (S.D.)	Min.	First quartile (Q1)	Median	Third quartile (Q3)	Max.
Capacitance 1 (pF)	saline	26.29	34.37	0.04	1.35	6.06	49.26	114.71
	brain	4.04	8.82	0.16	0.27	0.84	2.72	37.93
Capacitance 2 (pF)	saline	94.51	89.65	8.00	25.99	73.48	129.48	440.72
	brain	15.43	30.32	0.80	1.62	3.99	9.83	156.23
Resistance 1 (kΩ)	saline	1097.38	867.39	219.53	522.65	755.40	1381.44	4372.89
	brain	2145.97	1890.58	18.30	325.07	1803.71	3330.09	7420.84
Q of CPE 1 (nS)	saline	35.32	34.36	1.18	8.12	21.40	63.32	103.59
	brain	40.83	44.98	0.99	8.63	18.28	72.54	203.02
Alpha of CPE 1	saline	0.66	0.14	0.45	0.52	0.64	0.78	0.93
	brain	0.61	0.15	0.35	0.48	0.59	0.70	0.93
Q of CPE 2 (nS)	saline	22.73	29.29	1.49	3.58	12.89	24.54	116.18
	brain	12.24	17.41	1.10	2.00	3.97	15.21	80.65
Alpha of CPE 2	saline	0.63	0.13	0.39	0.53	0.61	0.74	0.87
	brain	0.59	0.27	-0.28	0.43	0.70	0.78	0.87
Resistance 2 (kΩ)	saline	5.79	1.90	1.23	4.31	5.63	7.30	11.41
	brain	222.97	390.26	13.70	43.37	61.08	138.63	1984.72
Capacitance 3 (pF)	saline	81.59	31.70	49.49	64.09	73.97	85.51	221.79
	brain	362.70	1245.44	8.35	13.00	15.13	67.68	7691.23

Table S4. Statistical report of the comparison between the fitted parameters in the saline and the pig brain model. Degree of Freedom = 1.

	χ^2-value	p-value
Capacitance 1	39.07325	4.08198E-10
Capacitance 2	21.8267	2.98418E-6
Resistance 1	4.25858	0.03905
Q of CPE 1	0.00901	0.92436
Alpha of CPE 1	2.90318	0.08841
Q of CPE 2	5.97011	0.01455
Alpha of CPE 2	0.02399	0.8769
Resistance 2	57.75949	2.96207E-14
Capacitance 3	15.85921	6.82336E-5
